# Eph-Ephrin Signaling Mediates Cross-Talk Within the Bone Microenvironment

**DOI:** 10.3389/fcell.2021.598612

**Published:** 2021-02-09

**Authors:** Agnieszka Arthur, Stan Gronthos

**Affiliations:** ^1^Mesenchymal Stem Cell Laboratory, Faculty of Health and Medical Sciences, Adelaide Medical School, University of Adelaide, Adelaide, SA, Australia; ^2^Precision Medicine Theme, South Australian Health and Medical Research Institute, Adelaide, SA, Australia

**Keywords:** bone marrow microenvironment, bone marrow mesenchymal stem cells, osteogenic differentiation, hematopoietic stem cells, osteoimmunology, vasculature, musculoskeletal pathology, Eph-ephrin communication

## Abstract

Skeletal integrity is maintained through the tightly regulated bone remodeling process that occurs continuously throughout postnatal life to replace old bone and to repair skeletal damage. This is maintained primarily through complex interactions between bone resorbing osteoclasts and bone forming osteoblasts. Other elements within the bone microenvironment, including stromal, osteogenic, hematopoietic, endothelial and neural cells, also contribute to maintaining skeletal integrity. Disruption of the dynamic interactions between these diverse cellular systems can lead to poor bone health and an increased susceptibility to skeletal diseases including osteopenia, osteoporosis, osteoarthritis, osteomalacia, and major fractures. Recent reports have implicated a direct role for the Eph tyrosine kinase receptors and their ephrin ligands during bone development, homeostasis and skeletal repair. These membrane-bound molecules mediate contact-dependent signaling through both the Eph receptors, termed *forward signaling*, and through the ephrin ligands, referred to as *reverse signaling*. This review will focus on Eph/ ephrin cross-talk as mediators of hematopoietic and stromal cell communication, and how these interactions contribute to blood/ bone marrow function and skeletal integrity during normal steady state or pathological conditions.

## Introduction - the Cellular Components of Bone Microenvironment

**Figure 1:**
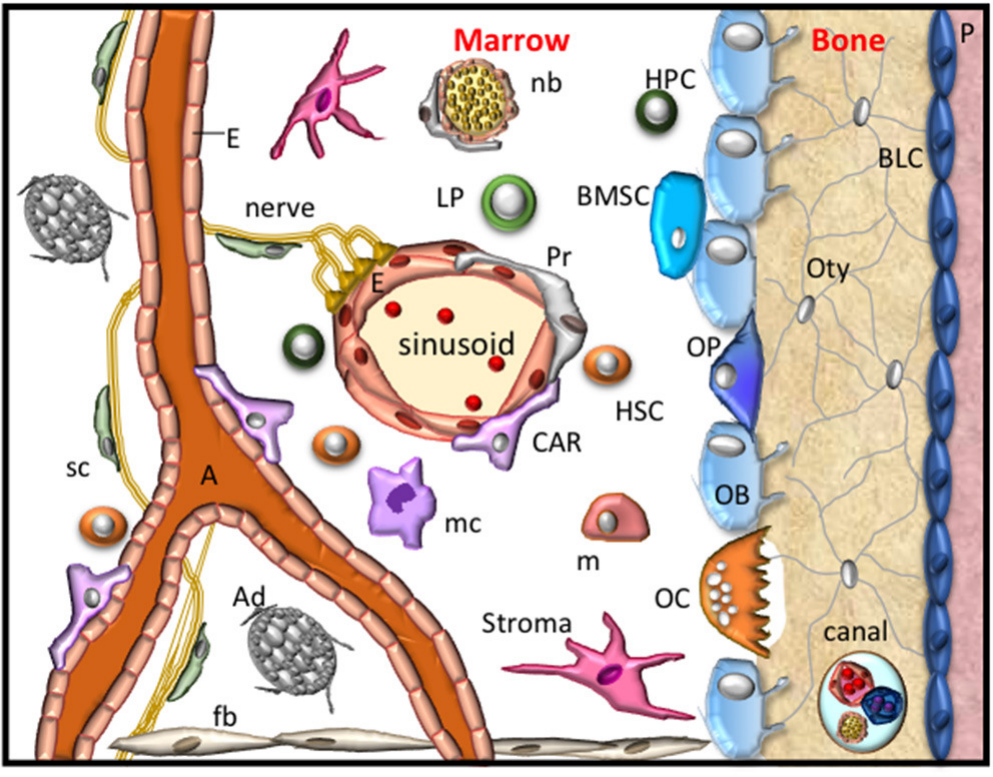
The bone microenvironment. A schematic representation of the resident cells within the bone microenvironment during homeostasis. These cellular components include fibroblasts (fb), stroma, bone marrow stem cells (BMSC), osteoprogenitors (OP), obsteoblasts (OB), bone lining cells (BLC), osteocytes (Oty), the periosteum (P), nerves, Schwann cells (sc), nerve bundle (nb), arterioles (A), endothelial cells (E), Pericytes (Pr), CXCL12-abundant reticular cells (CAR), also known as Leptin Receptor+ mesenchymal stromal cells, adipocytes (Ad), hematopoietic stem cells (HSC), hematopoietic progenitor (HPC) cells, lymphoid progenitors (LP), monocytes (m), macrophage (mc) and osteoclasts (OC). These cells form specific niches to regulate haematopoiesis and osteogenesis and thus maintain skeletal integrity.

The bone microenvironment provides cellular, molecular, and metabolic stimuli in an endocrine, paracrine and autocrine manner to regulate and maintain skeletal integrity, support hematopoiesis and regulate immune cell responses. The cellular components that reside within the bone microenvironment include endothelial cells, perivascular cells, neural cells, Schwann cells, and those of the mesenchymal and hematopoietic lineages. These populations contribute to specific stem cells niches located within the bone marrow and the bone to support and maintain hematopoiesis and osteogenesis (Figure 1) (Chan et al., 2015; Ramasamy et al., 2016; Crane et al., 2017). Hematopoiesis is sustained by hematopoietic stem cells (HSC) that give rise to the erythroid (erythrocytes, megakaryocytes, platelets), myeloid (basophil/ mast cells, eosinophils, neutrophils, dendritic cells, monocytes, macrophages and osteoclasts) and lymphoid (T-lymphocytes, B-lymphocytes and natural killer cells) lineages. Mesenchymal stem cells (MSC) give rise to cells of the chondrogenic lineage (chondroprogenitors, proliferating, resting, pre-hypertrophic and hypertrophic chondrocytes), osteogenic lineage (osteoprogenitors, osteoblasts, bone lining cells, osteocytes), stromal cells, reticular cells, smooth muscle cells and adipocytes (Figure 1). Maintenance of the bone microenvironment under physiological or pathological conditions is dependent on interactions between the different cellular components, as well as their precise anatomical location within the skeleton. However, a better understanding of the numerous molecular interactions that mediate intercellular signaling and function within the bone microenvironment is required to help identify novel therapeutic strategies to treat musculoskeletal conditions. The present review describes the erythropoietin-producing human hepatocellular (Eph) receptor tyrosine kinase family and the Eph receptor interacting protein (ephrin) ligands (also termed Efn molecules) that are expressed by stromal, hematopoietic, and vascular populations and the function of Eph/ephrin molecules within the bone microenvironment.

## Overview of the Eph-Ephrin Molecules

The Eph family of receptor tyrosine kinases (RTKs) and their ephrin ligands are contact-dependent, cell membrane-bound molecules expressed by invertebrates and vertebrate species. This family consists of two subclasses, the A subclass and the B subclass, comprising 14 Eph receptors (EphA1-8 and EphA10 and EphB1-4 and EphB6) and eight ephrin ligands (ephrin-A1-A5 and ephrin-B1-B3) in humans (Kania and Klein, 2016; Nguyen et al., 2016a; Liang et al., 2019). The Eph-ephrin intercellular and intracellular signaling modalities, both catalytic and non-catalytic, are complex. The structure of these molecules, the size of the family, and the range of activation-dependent downstream effects all contribute to this complexity (Gale et al., 1996; Kania and Klein, 2016; Nguyen et al., 2016a; Liang et al., 2019).

It is important to note that there is promiscuous binding within subclasses, where multiple EphA receptors bind with differing affinity to cognate ephrin-A ligands and EphB receptors bind with ephrin-B ligands. The Eph receptors of both subclasses are predominantly structurally conserved, with the extracellular region consisting of the globular ligand binding domain, a cysteine-rich region, encompassing the Sushi and epidermal growth factor like domains, and two fibronectin III repeats. Intracellularly, Eph receptors consist of a juxtamembrane, a kinase domain, a sterile alpha motif (SAM) domain and a postsynaptic density, discs-large, zona occludens-1 (PDZ) domain. The most variation within these receptors lies in the ligand binding domain and thus the receptors are functionally divided into two subclasses determined by the binding affinity for their cognate ligand (Gale et al., 1996), with minimal interaction between subclass, with the exception of EphA4 which can bind with ephrin-B ligands; and EphB2 which can also interact with ephrin-A5 (Gale et al., 1996; Holland et al., 1998; Himanen et al., 2004).

Conversely, the ephrin ligands are divided into their subclasses based on the variation in their structure. While both A and B subclass ligands contain the extracellular receptor binding globular domain, the ephrin-A molecules are glycosylphosphatidylinositol (GPI) linked to the exoplasmic leaflet of the plasma membrane (Pasquale, 2005). The ephrin-B molecules however are transmembrane molecules consisting of a transmembrane domain containing conserved tyrosine residues and a C-terminal PDZ domain-binding motif (Liang et al., 2019).

The Eph RTK family does not fall into the conventional receptor ligand signaling mechanism, where the terms “receptor” and “ligand” are somewhat artificial. Both the Eph and ephrin expressing cells are able to signal and thus can function as both receptors and ligands. Conventional signaling through the Eph receptor following ligand binding is referred to as *forward signaling*, while activation of an ephrin ligand upon Eph receptor binding is considered *reverse signaling* (Bruckner et al., 1997; Binns et al., 2000; Murai and Pasquale, 2003). Furthermore, the Eph-ephrin molecules can mediate their response uni-directionally, through either the Eph or ephrin expressing cell, or bi-directionally, through both Eph and ephrin expressing cells simultaneously as reviewed by Kania and Klein (2016). These interactions can be mediated in *trans*, where opposing cells express the receptor or ligand; or in *cis*, where both the receptor and ligand are expressed on the same cell (Dudanova and Klein, 2011; Falivelli et al., 2013; Yoshida et al., 2017). These receptors and ligands interact as dimers and tetramers and larger clusters, where the clustering of Eph and ephrin molecules is essential to provoke a specific response within a cell (Davis et al., 1994; Himanen et al., 2001; Xu et al., 2013; Liang et al., 2019). The biological outcomes such as adhesion, de-adhesion, migration, proliferation or differentiation are dependent on the quantitative characteristics of Eph activation, where high levels and low levels of expression/activation can induce opposing biological responses (Batlle et al., 2002; Blits-Huizinga et al., 2004; Hansen et al., 2004; Poliakov et al., 2004; Ojosnegros et al., 2017).

The Eph receptors of both subclasses and the ephrin-B ligands can signal through both tyrosine phosphorylation and ensuing protein-protein interactions, as well as protein-protein interactions through the PDZ motif and SAM domain (Binns et al., 2000; Cowan and Henkemeyer, 2001; Lu et al., 2001; Palmer et al., 2002; Leone et al., 2008; Wang et al., 2018; Liang et al., 2019; Baudet et al., 2020). Importantly the ephrin-A molecules localize to lipid rafts/micro-compartments within the plasma membrane and engage transmembrane proteins, such as caveolins, neurotrophin receptor p75 and intracellular Src family kinase dependent signaling (Davy et al., 1999; Davy and Robbins, 2000; Lim et al., 2008). The signaling through Eph-ephrin interactions is essential for a number of developmental and pathophysiological processes (Boyd et al., 2014; Kania and Klein, 2016), including cell attachment, spreading, migration, tissue boundary formation, cellular differentiation, stem cell niche maintenance and proliferation, axon guidance, neural plasticity, somatogenesis, angiogenesis, vasculogenesis, hematopoiesis, immune cell function, cancer tumorigenicity, tissue repair, skeletal development and homeostasis (Kullander and Klein, 2002; Cramer and Miko, 2016; Kania and Klein, 2016; Yang et al., 2018a; Darling and Lamb, 2019; Alfaro et al., 2020; Buckens et al., 2020; Fernandez-Alonso et al., 2020; Giorgio et al., 2020; Vreeken et al., 2020).

## The Role of Eph-Ephrin Signaling Within the Mesenchymal Lineage and Contribution to Skeletal Development and Homeostasis

**Figure 2:**
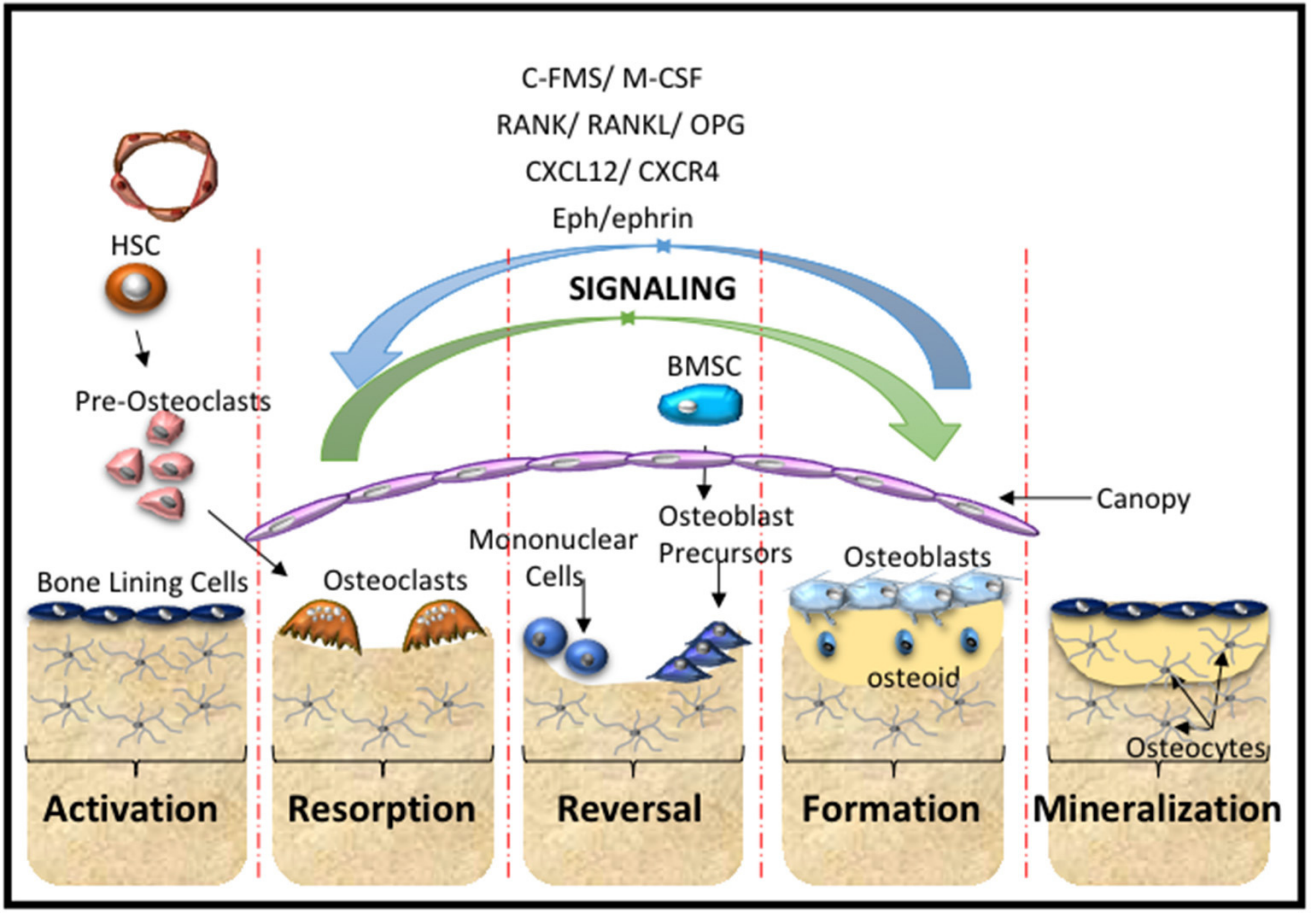
Bone Remodeling. A schematic overview of the cells and main molecular processes involved during the stages of bone remodeling starting with the activation stage, where hematopoietic stem cells (HSC) give rise to pre-osteoclasts of the myeloid lineage that are recruited to the injury site. This is followed by the resorption stage where the pre-osteoclasts undergo maturation and fusion to form mature multinucleated osteoclasts that resorb the bone matrix. The reversal stage sequesters bone marrow stromal stem cells (BMSC) and osteoblast progenitors to the repair site, which is followed by the formation stage, where the bone matrix is synthesized and osteoid is laid down. The mineralization stage involves the mineralization of the osteoid and regeneration of new bone. These cellular responses are mediated by molecular interactions and signaling cascades. The major contributors include the C-FMS, M-CSF, the RANK/RANL/OPG axis, CXCL12/CXCR4 signaling and Eph-ephrin communication.

The skeleton is formed through two distinct processes termed intramembranous and endochondral ossification. MSC are essential for both processes. Cranial neural crest and the direct differentiation of MSC into osteoblasts contribute to intramembranous ossification. A portion of the clavicle and the cranium are formed by intramembranous ossification. The more complex process of endochondral ossification contributes to the formation of posterior part of the skull, axial and appendicular skeleton. In simplistic terms, during endochondral bone formation MSC condensations contribute to the formation of a cartilaginous scaffold that is systemically replaced to form bone. However, this is a much more involved process which has been elegantly described by Kronenberg and colleagues (Ono et al., 2019). Skeletal integrity is maintained throughout the lifetime of vertebrates through the tightly regulated process of bone homeostasis which takes place within the basic multicellular unit (BMU) or bone remodeling unit (BRU). This process predominantly relies on many cell types maintaining the delicate balance between bone formation and bone resorption (Arthur et al., 2013b; Sims and Martin, 2014) (Figure 2).

### Identification and Maintenance of MSC Through Eph-Ephrin Signaling

MSC are a desirable source of cells to use in bone tissue engineering applications due to their accessibility, differentiation potential and immune-modulatory effects (Arthur et al., 2009b; Nguyen et al., 2013; Wada et al., 2013). Notably, numerous MSC populations that contribute to the skeletal stem cell niche have been identified using mouse *in vivo* studies (Chan et al., 2015; Crane et al., 2017). A comprehensive review of genetic mouse studies identifying the bone marrow stem cells niche and the translational relevance to human stem cell biology has been described (Chen et al., 2017). Human MSC populations are predominantly referred to as MSC or bone marrow stromal/stem cells (BMSC) and are defined based on three criteria proposed by The Mesenchymal and Tissue Stem Cell Committee of the International Society for Cellular Therapy. These include: (1) that isolated cells are plastic adherent in culture: (2) that >95% of the cells express the following markers CD73 CD90, and CD105, and >95% of the cells lack the expression of CD14 or CD11b, CD79a or CD19, CD34, CD45, and HLA-DR: and (3) that the cultured MSC have the ability to differentiate into osteoblasts, adipocytes and chondroblasts (Dominici et al., 2006). However, these criteria are an oversimplification of MSC-specific populations and are inadequate indicators of stemness. Other markers have been identified with the capacity to purify clonogenic MSC which exhibit multi-differentiation potential, hematopoietic support and self-renewal capacity *in vitro* and *in vivo*, based on their high cell surface expression of NGF-R, PDGF-R, EGF-R, IGF-R, CD49a/CD29, STRO-1, CD146, and CD106 (Gronthos and Simmons, 1995; Gronthos et al., 2001, 2003; Dennis et al., 2002; Shi and Gronthos, 2003; Sacchetti et al., 2007).

The Eph-ephrin molecules have also been implicated in MSC biology (Alfaro et al., 2020). Certainly comparative analyses studies have identified upregulated levels of EphA2 expression in umbilical cord MSC, compared to MSC derived from other tissue sources and human dermal fibroblastic cells, suggesting that EphA2 may be an unique biomarker characterizing tissue specific MSC (Brinkhof et al., 2020). It is important to note that during cell culture a number of MSC biomarkers are downregulated rapidly coincident with a correlative increase in expression of osteogenic maturation associated genes (Gronthos et al., 2003). While the expression of EphA3 in endometrial MSCs can be dependent on oxygen levels during culture conditions (To et al., 2014). Furthermore, cell passage and cellular aging also contribute to the ability of MSC to adequately differentiate toward the osteogenic lineage (Tanabe et al., 2008). This is an important issue with respect to tissue engineering, which requires clinical grade and scale up of MSC numbers for therapeutic applications. Multiple groups have endeavored to address this issue by investigating the differential gene expression profile of short- and long-term passaged human BMSC (hBMSC), identifying EphA5 among other molecules to be up-regulated during late passages (Tanabe et al., 2008; Yamada et al., 2013). It was proposed that EphA5 mediates inhibitory signals observed in long-term cultures that led to the deterioration of hBMSC differentiation capacity. Therefore, EphA5 may be a negative regulator of hBMSC osteogenic differentiation (Yamada et al., 2013). Subsequent overexpression and siRNA studies support this role for EphA5 and further propose that EphA5 signaling may have a dual role in growth regulation of hBMSC and may also be a potential candidate for replicative senescence (Yamada et al., 2016). Indeed, it was recently proposed that members of both the A and B subclass Eph-ephrin molecules are able to influence MSC survival and adherence *in vitro* (Alfaro and Zapata, 2018).

The B-subclass also contribute to MSC-like populations derived from dental and bone marrow tissues (Stokowski et al., 2007; Arthur et al., 2009a, 2011). Numerous EphB-ephrin-B molecules are expressed by human dental pulp stem cells (hDPSC) within the perivascular niche and the surrounding tissue. Both EphB and ephrin-B molecules play a functional role regulating cell attachment and spreading, and inhibiting cell migration (Stokowski et al., 2007). In the context of an *ex vivo* tooth injury model it was further confirmed that ephrin-B1 activation of EphB molecules expressed by hDPSC was important for MSC niche maintenance under steady-state conditions (Arthur et al., 2009a). Similarly, the B-subclass have also been identified in hBMSC where EphB-ephrin-B communication mediated through *reverse signaling* inhibited hBMSC attachment and spreading, while *forward signaling* promoted migration (Arthur et al., 2011). *Reverse signaling* through ephrin-B molecules is also important for hBMSC chondrogenic and osteogenic differentiation (Arthur et al., 2011). Collectively, these findings demonstrate the importance of Eph-ephrin communication in MSC niche maintenance and differentiation capacity in response to injury of mineralized tissues.

### Contribution of Eph-Ephrin Signaling to Chondrogenesis

**Figure 3:**
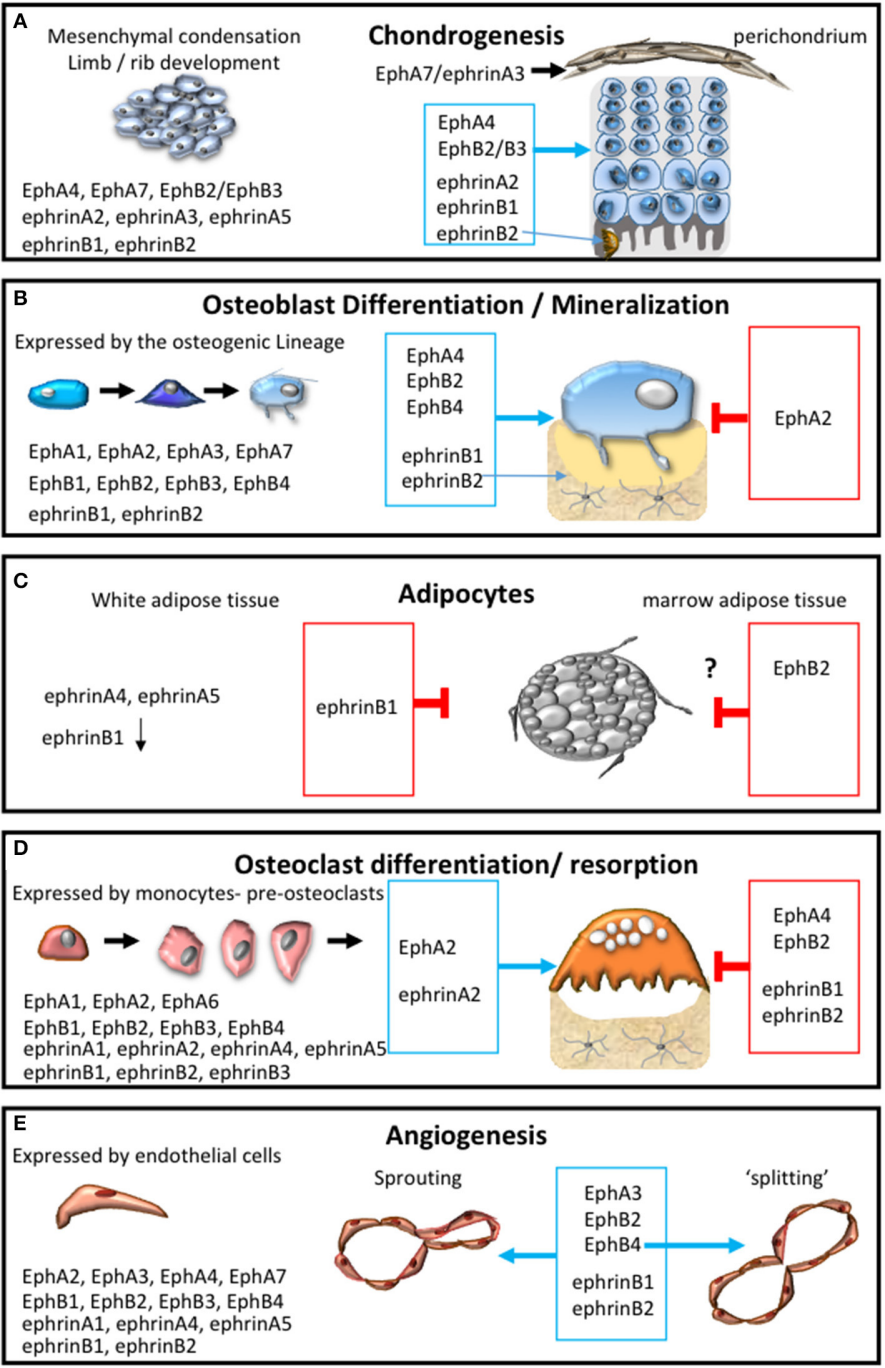
Eph-ephrin communication within the bone microenvironment. The expression profile of A and B subclass Eph and ephrin molecules and their influence on: **(A)** chondrogensis and cartilage formation; **(B)** osteogenic differentiation and mineral formation, **(C)** adipocyte function within white and marrow adipose tissue; **(D)** osteoclast formation, differentiation and resorptive function; **(E)** during the processes of angiogenesis including adhesion, migration, sprouting and intussusceptive “splitting” angiogenesis. The permissive signal is represented in the blue box and the inhibitory response is represented in the red box.

Pioneering studies have identified the importance of spatial localization of EphA-ephrin-A signaling (EphA4, EphA7, ephrin-A2, ephrin-A3, ephrin-A5) within the earliest stages of skeletal development (Wada et al., 1998, 2003; Stadler et al., 2001; Lorda-Diez et al., 2011) (Figure 3A). During the early stages of endochondral ossification, the outer cells of the mesenchymal condensation form the perichondrium, which display overlapping expression of ephrin-A3 and EphA7. EphA7, positively regulated by Hoxa13, subsequently communicates with ephrin-A3 to demarcate the perichondrial boundary (Stadler et al., 2001). Within the developing avian limb bud, ephrin-A2, localized predominantly to the proximal-intermediate regions, regulates the “position-specific” affinity of limb mesenchymal cells, while also contributing to cartilage patterning within the limb (Wada et al., 2003). Whilst, Eph-ephrin communication is critical for spatial localization, down-stream EphA4 forward signaling has also been shown to contribute to post-natal body growth through the regulation of insulin growth factor (IGF-1). Thus global deletion of EphA4 results in smaller epiphyseal growth plates and short stature and associated low levels of plasma IGF-1 (Jing et al., 2012). The EphA receptors have also been identified within the superficial to middle zone during articular cartilage growth using laser capture microdissection. However, this study did not elaborate further on which receptors were differentially expressed (Lui et al., 2015). Several of these molecules are now being addressed in the context of cartilage related defects, which will be discussed in Section Pathological Conditions Attributed to Alterations Within the Bone Microenvironment in Response to Eph-Ephrin Function of the review.

Recently, the B-subclass of Eph-ephrins has also been implicated in the growth of articular cartilage (Lui et al., 2015). The ephrin-B1 molecule is the only known family member to be associated with a human skeletal phenotype. In humans, loss of function mutations in the *EFNB1* gene result in cranial defects such as frontonasal dysplasia and coronal craniosynostosis (Twigg et al., 2004; van den Elzen et al., 2014). Associated skeletal defects include asymmetrical lower limb shortness and unequal arm span to total height ratio (van den Elzen et al., 2014), which are dependent on correct chondrogenesis and growth plate function. Polydactyly, a cartilage segmentation defect, was also observed in humans (Wieland et al., 2004). The global deletion of ephrin-B1 in mouse causes perinatal lethality and other defects including abnormal cartilage segmentation, ossification pattern (Compagni et al., 2003); and perichondrium maintenance (Davy et al., 2004). Furthermore, abnormalities in cartilage segmentation within the wrist and ribs during embryonic development and in adult ephrin-B1 null mice have also been described (Compagni et al., 2003; Davy et al., 2004). More recently, ephrin-B1 was also identified to be important for growth plate formation (Figure 3A). The targeted deletion of ephrin-B1 under the control of the *Osterix* promoter (*Osx:cre-eB1*^−/−^*)* resulted in developmental growth plate defects in *Osx:cre-eB1*^−/−^ mice when compared to *Osx:cre* controls (Nguyen et al., 2016b). Notably, osterix is expressed by pre-hypertrophic chondrocytes within the growth plate as well as osteogenic progenitors (Oh et al., 2012). In accord with these observations, *in vitro* studies have also noted that ephrin-B1 *reverse signaling* enhances the chondrogenic potential of hBMSC (Arthur et al., 2011), where it contributes to the regulation of the fracture repair process (Arthur et al., 2020).

Interestingly, it appears that the loss of ephrin-B2 also under the control of the *Osterix* promoter, resulted in a strikingly different phenotype. These *Osx1Cre:Efnb2*^Δ/Δ^ mice displayed an increase in trabecular bone volume, growth plate remnants and abnormal osteoclasts within the growth plate during skeletal development, which was resolved by 6 weeks of age (Tonna et al., 2016). This observation was attributed to the dependence of ephrin-B2 signaling for the correct production of cartilage degrading enzymes and subsequent endochondral ossification, which then allowed for the correct attachment of osteoclasts and also osteoblasts to the chondro/osseous junction (Tonna et al., 2016) (Figure 3A). During postnatal bone development of the secondary ossification center, IGF-1 signaling within the inner layer of perichondral cells promotes proliferation and cartilage matrix degradation (Kozhemyakina et al., 2015). Moreover, IGF-1 increased ephrin-B2 production, which stimulated VEGF expression and subsequent vascularization (Wang et al., 2015). Notably the conditional deletion of ephrin-B2 under the control of the Collagen Type 2 promoter, which has been proposed through lineage tracing studies to be expressed prior to Osterix (Ono et al., 2014), did not result in obvious growth plate defects. Rather these conditional ephrin-B2 knockout mice displayed a defect in the trabecular bone in the metaphysis and epiphysis (Wang et al., 2020). The authors propose that during skeletal development ephrin-B2 expressed by Collagen Type 2 expressing cells contribute to the transdifferentiation of chondrocytes to osteoblasts (Wang et al., 2020). This suggests that in addition to normal endochondral ossification, a proportion of chondrocytes can transdifferentiate into osteoblasts within ossification centers. This is a relatively new area of investigation that is gaining momentum (Yang et al., 2014; Zhou et al., 2014); although the concept still requires further examination. However, it is clear that while ephrin-B1 and ephrin-B2 are structurally similar their function can vary greatly depending on their spatial and temporal expression and interaction with cognate receptors (Figure 3A).

### Contribution of Eph-Ephrin Signaling to the Osteogenic Lineage

The seminal work conducted by Matsuo et al. in 2006 demonstrated that EphB4, expressed by osteoblasts, and its cognate ligand, ephrin-B2, expressed by osteoclasts signal bi-directionally acting as mediators of bone homeostasis (Zhao et al., 2006). This inspired a body of work examining the importance of Eph-ephrin communication during skeletal development, homeostasis and skeletal repair (Edwards and Mundy, 2008; Martin et al., 2010; Matsuo and Otaki, 2012; Sims and Walsh, 2012; Arthur et al., 2013a; Sims and Martin, 2014; Tonna and Sims, 2014; Rundle et al., 2016) (Figure 3B). The research in this field has predominantly focused on the B-subclass Eph-ephrin molecules and the communication between EphB4-ephrin-B2. However, a number of EphA molecules are also expressed within the osteogenic population, including EphA1, A2, A3, A4, and A7 (Zhao et al., 2006; Irie et al., 2009; Matsuo and Otaki, 2012; Stiffel et al., 2014). While EphA4 is important for limb development, chondrogenesis and cranial development, EphA2 has been directly implicated in osteogenic function, inhibiting osteogenesis through RhoA signaling (Zhao et al., 2006; Irie et al., 2009; Ting et al., 2009; Matsuo and Otaki, 2012; Stiffel et al., 2014).

#### EphB4-Ephrinb2 Communication Within Skeletal Tissue

Our understanding of EphB4-ephrin-B2 communication has expanded over the last 15 years from cell-heterotypic interactions between cells of the osteogenic lineage and osteoclastic cells to cell-homotypic interactions within the osteogenic lineage. The field has utilized the knowledge of Eph-ephrin function from other biological systems and their association with diverse signaling networks (Arvanitis and Davy, 2008), to understand the molecular interactions of cell surface signaling pathways within skeletal tissue (Lindsey et al., 2018). It is clear that EphB4 *forward signaling* is required for bone formation under steady-state (Zhao et al., 2006) and trauma induced conditions (Arthur et al., 2013a).

Mechanistically, inhibiting EphB4-ephrin-B2 interactions within the osteogenic population reduces the mineralization potential of mouse stromal cells in a dose dependent manner by parathyroid hormone 1 receptor (PTHR1) (Allan et al., 2008). These observations suggest that ephrin-B2-expressing osteogenic cells are responsive to parathyroid hormone-related protein (PTHrP)/ PTH mediating homotypic interactions presumably with EphB4 and potentially EphB2 to stimulate osteoblast maturation and function (Allan et al., 2008). The N-terminus of PTHrP has been attributed with roles in calcium homeostasis and osteogenic function among other roles. However, it is also evident from mouse knock-in studies that the mid-regional, nuclear localization sequence (NLS) and C-terminus of PTHrP are also essential for osteogenesis (Toribio et al., 2010). These regions influence skeletal mineralization in part through the regulation of ephrin-B2 within the osteogenic lineage (Toribio et al., 2010). Moreover, administration of PTH in the presence of EphB4 blocking peptide to inhibit EphB4-ephrin-B2 interactions, resulted in a multifaceted response in both osteoblasts and osteoclasts *in vitro* and *in vivo* (Takyar et al., 2013). Inhibition of EphB4 mediated signaling reduced the expression of mature osteoblast and osteocyte markers *in vitro*, while osteoblast numbers and activity were increased *in vivo* correlating to a decrease in trabecular number. Collectively the findings suggest that PTH mediated EphB4 *forward signaling* within the osteogenic lineage is important for the later phases of osteoblast differentiation (Takyar et al., 2013).

Furthermore, it is well established that IGF-1 signaling is necessary for PTH stimulation of bone formation (Bikle et al., 2002; Bikle and Wang, 2012). Indeed it has been demonstrated utilizing global IGF-1 knockout mice and complementary *in vitro* co-culture studies using blocking peptides that IGF-1/ IGF-IR signaling mediated through ephrin-B2-EphB4 heterotypic interactions promoted osteoblast and chondrogenic differentiation (Wang et al., 2015). However, while the majority of these studies have focused on EphB4 signaling during osteogenic differentiation, a recent report identified that ephrin-B2 *reverse signaling* is also important for secondary mineralization (Vrahnas et al., 2019). The bone is mineralized through two sequential phases, known as primary mineralization at the calcification front, which is a rapid process (~60–65% mineralization in ewes). This is followed by secondary mineralization, involving the gradual maturation, accumulation and quality of mineral (Bala et al., 2010). Assessment of an osteocyte specific ephrin-B2 conditional knockout mouse found that the mice developed brittle bones. This was attributed to an acceleration of secondary mineralization resulting in increased mineral and carbonate accrual mediated by enhanced autophagic flux (Vrahnas et al., 2019). This novel finding demonstrates that B-type Eph and ephrin molecules are required for various processes during osteogenesis.

#### EphB2-EphrinB1 Communication Within Skeletal Tissue

The EphB2 high affinity ligand, ephrin-B1, is expressed by different human MSC-like populations and is a potent mediator of mineralization in both dental (Arthur et al., 2009a) and bone tissues (Arthur et al., 2011), skeletal development (Xing et al., 2010; Nguyen et al., 2016b), homeostasis (Arthur et al., 2018) and trauma (Arthur et al., 2020). Mechanical loading is essential for the maintenance of skeletal integrity, mechanical loading experiments using the tibia identified up-regulation of both EphB2 and ephrin-B1 when compared to the un-loaded control (Xing et al., 2005; Kesavan et al., 2011). EphB2 up-regulation was exacerbated within newly formed bone of transgenic mice overexpressing ephrin-B1 in committed bone cells, suggesting homotypic cellular interactions (Cheng et al., 2013). Since EphB4 expression was unchanged in these studies, mineralization may occur through EphB2-ephrin-B1 interactions independent of EphB4-ephrin-B2 signaling. While EphB2 has been implicated in osteogenesis within the cranial sutures (Benson et al., 2012), it was reported, although not shown, that EphB2 global knockout mice did not develop noticeable differences within the skeleton (Compagni et al., 2003). However, EphB2/EphB3 knockout mice were reported to display patterning abnormalities in the thoracic skeleton (Compagni et al., 2003), indicating some level of functional redundancy within the family. A conditional osteogenic EphB2 knockout study is thus warranted to determine the specific role of EphB2 during axial and appendicular skeletal development and homeostasis.

More is known about the role of ephrin-B1 in osteogenesis, where the global and conditional knockout of ephrin-B1 in osteoblasts results in gross skeletal deformities (Compagni et al., 2003; Xing et al., 2010; Nguyen et al., 2016b). These conditional mice are physically shorter in stature which correlated to reduced bone formation, cortical thickness, and trabecular parameters (Xing et al., 2010; Nguyen et al., 2016b). Conversely, transgenic mice over-expressing ephrinB1 in osteoblast progenitors exhibit enhanced bone formation, within the trabecular and cortical bone, and reduced bone resorption, resulting in an increase in bone mass (Cheng et al., 2013). Importantly, aging (6-month-old) mice lacking ephrin-B1 in the osteogenic population developed an osteoporotic-like phenotype (Arthur et al., 2018). Interestingly, mice with ephrin-B2 knockout using the same promoter reported a significant increase in bone to tissue volume, trabecular number, and thickness at 6 months of age (Tonna et al., 2014). Therefore, it appears that the functions of ephrin-B1 and ephrin-B2 vary considerably during osteogenesis. It appears that the function of these ephrin-B molecules is underpinned by their intercellular interaction with cognate receptors, predominantly facilitated by EphB2 and EphB4, respectively, and subsequent differential intracellular signaling modalities.

Mechanistically, ephrin-B1 intracellular signaling contributes to bone formation in mouse osteogenic cells, mediated through the PDZ domain. The binding of EphB2 with ephrin-B1, results in ephrin-B1 phosphorylation, consequently the ephrin-B1 PDZ domain forms a complex with Protein Tyrosine Phosphatase Non-Receptor Type 13 (PTPN13), Na+/H+ exchanger regulatory factor 1 (NHERF1) and Transcriptional Coactivator With PDZ-Binding Motif (TAZ). TAZ is subsequently de-phosphorylated and released from the ephrin-B1-PDZ complex and translocates to the nucleus inducing the expression of *Osterix* to drive osteoblast maturation (Xing et al., 2010). Recently it was confirmed that hBMSC also utilize the same signaling pathway where EphB2 activation resulted in the de-phosphorylation of TAZ (Arthur et al., 2020).

### Involvement of Eph/Ephrin Molecules in Adipogenesis

It is interesting to note that no studies have investigated the contribution of Eph-ephrin signaling within bone marrow adipocytes specifically. However, Zapata et al. recently reported that adipose tissue derived MSC (Ad-MSC) isolated from mice lacking EphB2 increase adipogenesis with minimal influence on osteoblast differentiation. However, Ad-MSC isolated from mice expressing a truncated version of EphB2, which prevents forward Eph signaling while still allowing ephrin reverse signal, resulted in osteoblast differentiation (Alfaro et al., 2020). These observations suggest that perhaps EphB2 forward signaling is important for the inhibition of adipogenesis by MSC. Moreover, Eph-ephrin communication has been reported in white adipose tissue, where ephrin-A4 and ephrin-A5 were found to be a downstream signaling pathway to aldehyde dehydrogenase, which stimulates the development and innervation of white adipose tissue (Shen et al., 2018). Also, ephrin-B1 was identified to be down-regulated in mature adipocytes of obese mice and shown to suppress the adipose inflammatory response (Mori et al., 2013).

Collectively these studies demonstrate that ephrin molecules of both subclasses are implicated in adipocyte biology and therefore investigating the function of Eph-ephrin molecules within bone marrow adipocytes is warranted (Figure 3C). Furthermore, it is clear that intercellular Eph-ephrin signaling within the mesenchymal lineage can modulate diverse pathways and biological responses during specific stages of skeletal development and bone homeostasis. However, other resident cells within the bone such as those of the hematopoietic lineage also contribute to skeletal development and homeostasis.

## Eph-Ephrin Communication Influences the Hematopoietic System Within the Bone

The HSC niche associates with numerous cell types and location within the bone marrow (Crane et al., 2017). These HSC and their derivatives, the myeloid and lymphoid lineages, are maintained and regulated by the stromal population (Okamoto and Takayanagi, 2019; Tsukasaki and Takayanagi, 2019; Guder et al., 2020). The contribution of Eph-ephrin intercellular signaling between the stromal population and the regulation of these lineages, while important, they are beyond the scope of this review. However, HSC niche maintenance and osteoclast function, both of which are essential for the maintenance of skeletal integrity have been addressed.

### Regulation and Maintenance of Hematopoietic Stem/Progenitors by BMSC Through Eph-Ephrin Signaling

**Figure 4:**
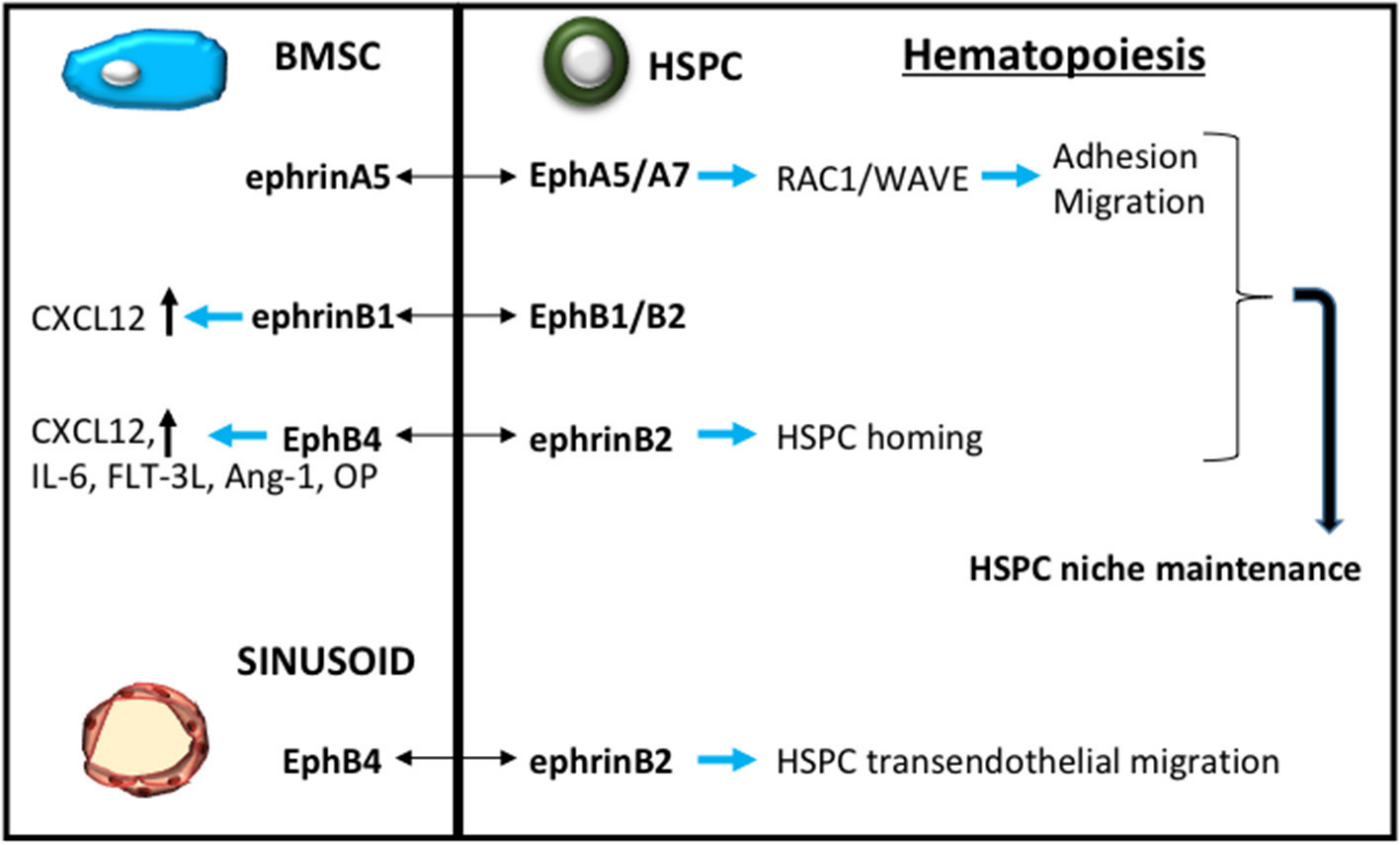
Eph-ephrin contribution to HSC niche maintenance. A schematic demonstrating Eph-ephrin signaling through bone marrow stromal stem cells (BMSC, blue) and sinusoid endothelium to regulate hematopoietic stem/progenitor cell (HPSC, green) maintenance and function.

We have previously reviewed the stromal–hematopoietic interactions through Eph-ephrin communication, highlighting the role of EphA3-ephrin-A5 and EphB4-ephrin-B2 interactions in BMSC-HSC intercellular signaling (Ting et al., 2010; Nguyen et al., 2015, 2016a). More recently, it has been reported that *EPHA5* and *EPHA7* are expressed by human hematopoietic stem/ progenitor cells. Activation of either EPHA5 or EPHA7 by EPHRIN-A5, expressed by the hBMSC, subsequently stimulates *RAC1* activation and RAC1 target molecule WAVE to enrich the maintenance, migration and adhesion of hematopoietic stem/ progenitor cells (Nguyen et al., 2017). The B-subclass act in a similar manner to the A-subclass in this BMSC-HSC intercellular communication. The conditional loss of ephrin-B1 within the mouse osteogenic population limits the capacity of these osteogenic cells to support the maintenance of mouse hematopoietic stem/progenitor cells (Arthur et al., 2019). Human studies confirmed that EPHB1 or EPHB2 expressing CD34^+^ hematopoietic stem/progenitor cells were responsive to ephrin-B1 stimulation (Arthur et al., 2019). Here it was proposed that the mechanism facilitating this response was mediated in part by *CXCL12* (Arthur et al., 2019), a known critical regulator of hematopoietic stem/progenitor cell function (Greenbaum et al., 2013) (Figure 4).

Further investigations into the contribution of EphB4-ephrin-B2 signaling in hematopoietic stem/ progenitor cell mobilization found that EphB4 was expressed by endomucin+ bone marrow sinusoidal endothelium, while ephrin-B2 was expressed by hematopoietic stem/progenitor cells, using a *EfnB2*^*H*2*BGFP*^ reporter mouse (Kwak et al., 2016). Importantly the study reported that the regulation of hematopoietic stem/progenitor cells exiting from the bone marrow was mediated through transendothelial migration, which could be inhibited by using antibodies that blocked EphB4-ephrin-B2 interactions. In the context of cancer therapy, blocking the mobilization of hematopoietic stem/ progenitor cell by inhibiting EphB4-ephrin-B2 communication also resulted in reduced infiltration of the hematopoietic stem/progenitor cells into murine tumor models (Kwak et al., 2016) (Figure 4). These observations suggest that manipulation of EphB-ephrin-B signaling has potential therapeutic applications not only in cancer but potentially other diseases and disorders.

### The Role of Eph/Ephrin Molecules in Osteoclast Development and Function

Seminal studies identified the expression and importance of a number of A and B subclass Eph receptors and ligands with discrete temporal functions within the osteoclast lineage (Zhao et al., 2006; Irie et al., 2009) (Figure 3D).

#### Contribution of the a Subclass Eph-Ephrin Molecules to Osteoclastogenesis

With regard to the EphA-ephrin-A molecules, osteoclast precursors were found to express EphA2 and ephrin-A2, while EphA4 is specifically expressed by mature osteoclasts. Further investigations revealed that EphA2 and ephrin-A2 are both positive regulators of osteoclast differentiation, where ephrin-A2 mediates down-stream signaling dependent on c-Fos, but not its target molecule NFATc1 (Irie et al., 2009). While it was proposed that ephrin-A2 *reverse signaling* may modulate intracellular calcium signaling through phospholipase Cγ2 (PLCγ2), further investigations are required to confirm these observations. Interestingly, ephrin-A2 was found to be cleaved by matrix metalloproteinases (MMPs), where its release enhanced osteoclastogenesis, suggestive of a homotypic interaction between ephrin-A2-EphA2 within the osteoclast lineage. However, as EphA2 was down-regulated when ephrin-A2 was up-regulated, it is plausible that this homotypic interaction takes place between osteoclasts at various developmental states (Irie et al., 2009).

Conversely, EphA4 expression by mature osteoclasts coincides with its function as a negative regulator of osteoclast activity rather than osteoclast formation (Stiffel et al., 2014). Assessment of EphA4-null mice showed reduced trabecular bone volume attributed to osteoclast size and resorption capacity with no change in osteoclast numbers. The molecular mechanisms facilitating this process are thought to be mediated through the activation of the β3-integrin signaling pathway leading to Vav3 activation (Stiffel et al., 2014), where Vav3 is a Rho family GTP exchange factor essential for actin cytoskeletal organization and resorptive activity (Faccio et al., 2005). Like the observations presented for the B-subclass during chondrogenesis and osteogenesis, here we also observe during osteoclastogenesis that EphA receptors, while structurally similar, disseminate diverse functional responses.

#### Contribution of the B Subclass Eph-Ephrin Molecules to Osteoclastogenesis

Initial studies identifying Eph-ephrin molecules during osteoclast differentiation did not detect the expression of EphB receptors within mouse osteoclast populations (Zhao et al., 2006). However, a recent study identified that *EPHB2* is expressed by human peripheral blood mononuclear cells and during osteoclast differentiation (Arthur et al., 2018). EPHB2 acts as a negative regulator of osteoclast differentiation and function *in vitro*, inhibiting TRAP^+^ osteoclast formation, resorption activity and the expression of *C-FMS, CXCR4, RANK*, and *CATHEPSIN K* (Arthur et al., 2018).

Conversely, the ephrin-B1 and ephrin-B2 ligands are expressed by mouse osteoclast progenitors and mature osteoclasts (Zhao et al., 2006). Loss-of-function studies determined that ephrin-B1 expressed by the myeloid lineage was a negative regulator of osteoclast differentiation (Cheng et al., 2012). It was proposed that EphB2 activation of ephrin-B1 inhibits NFATc1 expression, while also reducing the phosphorylation of ezrin/ radixin/ moesin (ERM) proteins in mature osteoclasts (Cheng et al., 2012). This protein complex is involved in cytoskeletal rearrangement and cell migration, which are important not only for osteoclast formation but also function. These observations suggest that ephrin-B1 *reverse signaling* plays an essential role for multiple processes in osteoclast biology.

It has also been shown that ephrin-B2 activation in osteoclast progenitors following EphB4 engagement suppresses osteoclast differentiation. This was mediated via the PDZ domain of ephrin-B2, which led to the inhibition of the osteoclastogenic c-Fos-NFATc1 cascade (Zhao et al., 2006; Mao et al., 2011; Wang et al., 2014). Interestingly, *in vitro* mouse osteoclast studies, in which titanium wear particles increased osteoclast formation and function showed that osteoclast activation and the expression of inflammatory markers could be attenuated with the addition of soluble EphB4-Fc, which binds and blocks the receptor binding domain of ephrin-B2 expressed by osteoclasts (Ge et al., 2018). This observation is of particular interest clinically as wear particles can induce inflammation and subsequent periprosthetic osteolysis in response to aseptic loosening following joint replacement surgery.

The communication between osteoblasts and osteoclasts is well established. It was recently documented that during skeletal development and aging (6 months old mice), mice lacking ephrin-B1 within osteoprogenitors displayed elevated osteoclast numbers within the secondary spongiosa and cortical bone (Nguyen et al., 2016b; Arthur et al., 2018). However, the lack of ephrin-B1 by osteoblasts, did not result in alterations in osteoclast numbers or function (Xing et al., 2010). These studies suggest that osteogenic progenitors also influence the function of the osteoclastic population. This observation is somewhat juxtaposed to current dogma which proposes that osteoblasts and osteocytes, rather than immature osteogenic populations, regulate osteoclast function (Han et al., 2018). However, immature osteogenic regulation of osteoclast function is also supported by the finding that administration of the EphB4 blocking peptide during PTH treatment enhanced osteoclast function *in vivo* (Takyar et al., 2013). Supportive evidence showed that the response was attributed to an indirect function of EphB4 signaling, where blocking of EphB4 in undifferentiated stromal Kusa 4b10 cells resulted in elevated levels of *Rankl, IL-6* and *Osmr*, known promoters of osteoclast formation (Takyar et al., 2013). Collectively, these studies imply that numerous Eph-ephrin interactions contribute to osteoclast function through distinctive spatially and temporally controlled molecular mechanisms. Further investigations are required to determine whether targeting Eph or ephrin molecules is an appropriate therapeutic approach to treat musculoskeletal conditions that are affected by the dysregulation of osteoclasts.

## The Role of Eph-Ephrin Signaling in Vascularization and Angiogenesis Within the Bone Microenvironment

Endothelial cells form blood vessels, supplying the skeletal tissue with nutrients, hormones, oxygen and growth factors, and are critical to skeletal growth, homeostasis and repair (Peng et al., 2020; Zhao and Xie, 2020). A recent review has highlighted the involvement of Eph-ephrin signaling in different endothelial cell populations (Vreeken et al., 2020), with few studies investigating the role of Eph-ephrin homotypic and heterotypic communication between mesenchymal and endothelial cells, during vascularization (formation of the vasculature), angiogenesis (expansion and remodeling of the vasculature) and capillary formation (Adams et al., 1999; Adams and Klein, 2000; Salvucci and Tosato, 2012). The B-subclass Eph-ephrin molecules have predominantly been implicated in these processes, where EphB4 and ephrin-B2 null mice are embryonically lethal (Wang et al., 1998). More specifically EphB3, EphB4 and ephrin-B1 are located on veins, while ephrin-B1 and ephrin-B2 are detected on arteries, where ephrin-B2 has also been implicated in arterial vasodilation (Stein et al., 1998; Adams et al., 1999; Gerety et al., 1999; Adams and Klein, 2000; Lin et al., 2014).

Endothelial cells and the mesenchyme express numerous Eph receptors and ephrin ligands that act through both homotypic and heterotypic interactions (Figure 3E). Vascular structures are also supported by pericytes, otherwise known as mural cells. Pericytes, identified by the perivascular marker CD146, share similar properties to MSC (Covas et al., 2008). These pericytes reside within the basement membrane of the vasculature and are key regulators of vascular maintenance and function through the secretion of angiogenic promoting factors. DPSC located within the perivascular niche have been shown to promote angiogenesis via the secretion of VEGF ligands, stimulating VEGFR2-dependent signaling pathways, which included the activation of ephrin-B2 (Janebodin et al., 2013). Activation of ephrin-B2, through its PDZ domain, has also been shown to control VEGFR2 and VEGFR3 endocytosis and subsequent angiogenic sprouting, lymphangiogenic growth and tumor angiogenesis (Sawamiphak et al., 2010; Wang et al., 2010). In the context of Eph-ephrin signaling the assembly of pericyte-endothelial cordlike structures (Figure 1), required for vascularization or remodeling, are reliant on Src phosphorylation-dependent down-stream signaling of ephrin-B2 in endothelial cells following activation by either EphB2 or EphB4 (Salvucci et al., 2009).

Similarly, homotypic communication between endothelial cells promotes the formation of cordlike structures, although this was mediated through EphB2 and EphB4 *forward signaling*, and enhanced CXCL12 endothelial chemotaxis (Salvucci et al., 2006). Endothelial cell migration and angiogenesis can also be facilitated by ephrin-B2 stimulation of EphB receptors and more specifically activating the phosphatidylinositol-3 kinase (PI3 kinase) pathway (Maekawa et al., 2003). Conversely, neovascularization can be facilitated by EphB1 stimulation of ephrin-B1 *reverse signaling*, mediated through the C-terminus and most likely the PDZ domain, and required for endothelial attachment and migration facilitated by integrin αvβ3 and α5β1 (Huynh-Do et al., 2002). Taken together, the processes of endothelial migration, angiogenesis and vascularization utilize both Eph forward and ephrin reverse signaling which appears to be dependent on intercellular communication.

Interestingly, EphB4 has also been identified as an important regulator of intussusceptive angiogenesis (splitting of blood vessels), a dynamic process of non-sprouting angiogenesis. Here it was shown that EphB4 can regulate dose-dependent outcomes of VEGF distribution to skeletal muscle that influenced ERK1/2 signaling down-stream of VEGFR2 to “fine tune” endothelial proliferation and circumferential enlargement of vessels without interfering with normal angiogenesis and endothelial migration (Groppa et al., 2018). While EphB4-ephrin-B2 communication is important for pericyte-mediated angiogenesis, this communication did not influence pericyte recruitment (Groppa et al., 2018). Notably, this process of intussusceptive angiogenesis was shown in the muscle, however intussusceptive angiogenesis has been reported in skeletal development and implicated in tumor growth (De Spiegelaere et al., 2012). As developmental processes are often recapitulated during repair, investigating intussusceptive angiogenesis following trauma or musculoskeletal disorders may provide new insight on the endothelial contribution of maintaining skeletal integrity.

In the context of the bone microenvironment, Eph-ephrin signaling of both subclasses has been implicated in tumor progression facilitating several processes including cell proliferation, migration, boundary formation and angiogenesis. More specifically, *EPHA3*, which is elevated in both bone marrow endothelial cells and plasma cells from Multiple Myeloma patients, promotes their adhesion, migration, angiogenesis and invasion (Caivano et al., 2017; La Rocca et al., 2017). Therefore EphA3 may be an appropriate target for the treatment of Multiple Myeloma (Caivano et al., 2017; La Rocca et al., 2017). Furthermore, endoglin-expressing endothelial cells were recently identified in the bone marrow during fetal development and during regeneration of the adult bone marrow following insult. It was proposed that these endothelial cells may contribute to angiogenesis, osteogenesis and hematopoiesis through the activation of “angiocrine factors.” While IL-33 was predominantly investigated in this study, EphA and EphB molecules were enriched in transcriptome studies of the fetal human regenerative endothelial cells (Kenswil et al., 2018).

With respect to tissue regeneration, bone marrow derived endothelial cells are a desirable source of cells that can promote angiogenesis and tissue repair. It was recently demonstrated through a tissue engineering strategy that modulating the stiffness of fabricated substrates regulated arterial-venous differentiation of bone marrow derived endothelial cells, where the EphB4 venous marker and ephrin-B2 arterial marker were differentially expressed based on substrate stiffness (Xue et al., 2017). Collectively, these studies demonstrate the importance of Eph-ephrin signaling in discrete biological processes to facilitate correct angiogenesis and vascularization. However, there is limited knowledge on the contribution of Eph-ephrin interactions within the endothelial population in postnatal skeletal tissues under steady state or pathological conditions or following trauma, warranting further investigation.

## Pathological Conditions Attributed to Alterations Within the Bone Microenvironment in Response to Eph-Ephrin Function

Eph-ephrin communication facilitates numerous processes within the bone marrow microenvironment that contribute to maintaining skeletal integrity. Therefore, the loss of any one of these signaling cascades can have detrimental effects to skeletal pathophysiology. While this review does not focus on the pathophysiology of skeletal malignancies, the Eph-ephrin molecules have been identified and contribute to numerous bone related cancers (Buckens et al., 2020).

### Osteoporosis

With an increasing aging population comes an increase in the frequency of bone related diseases such as osteoporosis, which is defined as “a systemic skeletal disease characterized by low bone mass and microarchitectural deterioration of bone tissue leading to enhanced bone fragility and a consequent increase in fracture risk,” according to The International Osteoporosis Foundation. Certainly, several studies using hormonally regulated osteoporotic ovariectomy (OVX)-induced bone loss models or similar models, have demonstrated the contribution of a number of the Eph-ephrin family members. One proposed treatment target is the communication between EphA2-ephrin-A2, where administration of 17β-estradiol following OVX in rats mitigated the associated bone loss partially through the suppression of EphA2-ephrin-A2 (Liu et al., 2018). Furthermore, an age-related model of osteoporosis in rhesus monkeys identified a gradual increase in bone mass following 12 weeks of treatment with miRNA-based gene therapy (miR-141). miR-141 targeted the osteoclast population, with no differences observed within several organs that were investigated (heart, liver, spleen, kidney) or metabolic processes (blood glucose or cholesterol levels). The study also identified that miR-141 could functionally target EphA2 within the osteoclast population (Yang et al., 2018b).

Prolonged use of glucocorticoids increases the incidence of osteoporotic fractures. The glucocorticoid-induced osteoporosis mouse model causes down-regulation of EphB4 in osteoblasts and up-regulation of ephrin-B2 in osteoclasts. This response was reversed following the administration of icariin, isolated from the Chinese herb Epimedium. Notably there were significant improvements in bone parameters following 4 weeks of icariin treatment (Huang et al., 2020). EphB4-ephrin-B2 expression is also dysregulated in a diabetes-related osteoporosis model (Wu et al., 2016). Together these observations demonstrate the importance of EphB4-ephrin-B2 intercellular communication in maintaining skeletal integrity. However, in the context of lactation-induced maternal bone loss, where prolactin is known to induce the release of osteoclast driver RANKL, up-regulation of the ephrin-B1 gene was detected, with no change in the expression of ephrin-B2 nor EphB4 (Wongdee et al., 2011). Importantly, the loss of ephrin-B1 within the osteogenic population alone results in an osteoporotic phenotype which is comparable to that observed in OVX-induced osteoporosis. Notably, the conditional loss of ephrin-B1 within the osteogenic population diminished skeletal integrity by attenuating bone formation and enhancing osteoclast numbers and function, which was mediated through EphB2 *forward signaling* (Arthur et al., 2018). This observation, in conjunction with its role in inhibiting osteoclast differentiation, suggests that ephrin-B1 may be a key driver in maintaining skeletal integrity. This is supported by the observation that administration of Alendronate (a bisphosphonate) for 8 weeks enhances the expression of ephrin-B1, EphB1 and EphB3 in the mouse femur. Based on *in vitro* cultures ephrin-B1 was also the highest expressed molecule on both osteoblasts and osteoclasts (Shimizu et al., 2012).

### Skeletal Repair

The Eph-ephrin molecules have also been investigated in the context of skeletal repair. Tazaki et al. addressed the bone remodeling process using the autologous transplant of goldfish scales, where the scales are formed by intramembranous ossification and mimic the bone remodeling process (outlined in Figure 2). While the data showed considerable variability between donors, the authors suggest that ephrin-B2-EphB was required for the activation of osteoprogenitor proliferation during the first absorption phase. During the formation phase ephrin-EphA4 signaling inhibited the communication between osteoclasts, while ephrin-B2 reverse signaling and EphB forward signaling were involved in osteoblast activation (Tazaki et al., 2018).

Stabilized femoral fracture studies have highlighted the importance and similar function of EphB4 and ephrin-B1 during the callus formation stage of bone modeling. More specifically the transgenic mouse studies demonstrated that EphB4 overexpression in committed bone cells facilitates callus formation *in vivo* following traumatic injury by enhancing endochondral ossification, while inhibiting osteoclast differentiation (Arthur et al., 2013a). Conversely, the loss of ephrin-B1 within the osteogenic lineage resulted in a delay in callus formation and skeletal repair, with an altered distribution of osteoclasts favoring the calcified cartilage (Arthur et al., 2020). This observation was consistent with an independent femoral fracture study that also suggested the importance of ephrin-B1 during the early stages of fracture repair, with the upregulation of ephrin-B1 at 1 and 2 weeks post fracture and localization with mature chondrocyte marker Collagen type 10 (Kaur et al., 2019).

Notably, ephrin-B2 was also upregulated during the first 3 weeks of fracture repair following a stabilized femoral fracture model, with its protein expression localizing to prehypertrophic chondrocytes, osteoblasts and osteocytes (Kaur et al., 2019). This is an interesting observation as it was recently reported that conditional loss of ephrin-B2 in Collagen Type 2 expressing cells also resulted in a significant delay in fracture repair. However, this study used a nonstable tibia fracture model (Wang et al., 2020). Using this model the authors demonstrated that loss of ephrin-B2 within the Collagen Type 2 expressing cells impaired intramembranous bone formation during fracture repair due to the decline in periosteal progenitors. The loss of ephrin-B2 also impaired endochondral ossification during fracture repair due to the reduction in progenitors and VEGF-induced vascular formation within the periosteum and the invasion front of the callus. In addition, there was a reduction in chondrocyte and osteoblast differentiation within the callus which led to impaired bone formation during the later stages of bone repair (Wang et al., 2020). Furthermore, the authors postulate a role for chondrocyte transformation to osteoblasts, although this requires further investigation. Interesting the formation or function of chondroclasts or osteoclasts was not investigated in this study, which is an essential aspect of endochondral bone formation. Collectively these studies demonstrate the relevance and importance of Eph-ephrin function during chondrogenesis and osteogenesis during skeletal repair. As such, targeting these molecules may be a viable therapeutic approach to treating skeletal trauma.

### Osteoarthritis

Osteoarthritis (OA) is a degenerative and debilitating disease of the joints and is the most common form of arthritis. While the etiology is not fully known, both systemic (obesity) and non-modifiable (gender, age, genetics) risk factors influence the progression of OA. The predominant feature of OA is the irreversible degradation of the structural proteins (collagens, proteoglycans) within the cartilage matrix of the articular cartilage, as well as cell death (Heinegard and Saxne, 2011). This loss of tissue results in cartilage thinning between adjacent bones, causing bone erosion, and in conjunction, the subchondral bone is remodeled causing sclerosis. Osteophyte formation (bone spurs) ensues, initially as cartilage outgrowths, which then undergo the developmental process of chondrogenesis/endochondral ossification (Hashimoto et al., 2002).

EphB4-ephrin-B2 communication has also been implicated in both chondrogenic and osteogenic metabolism following OA. In OA patients *EPHB4* gene expression is up-regulated in chondrocytes and in osteoblasts of the subchondral bone, where these osteoblasts have pro-resorption properties (Kwan Tat et al., 2008, 2009). Treatment of these chondrocytes or osteoblasts with ephrin-B2 *in vitro* reduced the expression of catabolic collagen degrading molecules in both the chondrocytes and osteoblasts, and inhibited the resorption activity of the osteoblasts (Kwan Tat et al., 2008, 2009). Furthermore, over-expression of EphB4 within osteoblasts was protective against cartilage degradation, sclerosis of the subchondral bone (Valverde-Franco et al., 2012) and the synovial membrane thickness in mice that had undergone the destabilization of the medial meniscus (DMM) OA model (Valverde-Franco et al., 2015). Interestingly, the loss of ephrin-B2 by chondrocytes instigated an osteoarthritic phenotype with aging alone (Valverde-Franco et al., 2016). Of note, ephrin-B1 has been implicated during the inflammatory processes of rheumatoid arthritis (Kitamura et al., 2008; Hu et al., 2015).

Novel approaches to investigate protein-protein interactions and associations between microRNA and genes are also being utilized to identify disease related targets for OA through the analysis of OA meniscal cells rather than the articular cartilage (Wang et al., 2013). The meniscus is composed of a heterogeneous extracellular matrix and fibroblast-like cells, chondrocyte-like cells, and cells with fusiform morphology (Makris et al., 2011). Among other molecules EphA4 was identified and associated with OA (Wang et al., 2013). Recently it was shown that EphA4 was expressed by articular chondrocytes, osteoblasts, osteocytes, meniscal and synovial cells within injured joints of an intraarticular knee injury model (Stiffel et al., 2020). Supportive *in vitro* studies demonstrated that ephrin-A4 stimulation of EphA4 mediated a pro-anabolic response within articular chondrocytes. While, EphA4 activation within synoviocytes facilitated an anti-catabolic response, the authors suggest that targeting EphA4 signaling may be a potential therapeutic approach to treat OA (Stiffel et al., 2020).

## Therapeutic Targets of Eph-Ephrin Signaling

The role of Eph-ephrin molecular interactions and specific signaling modalities in numerous tissues and related cancers has led to the development of multiple therapeutic approaches and targets (Barquilla and Pasquale, 2015; Buckens et al., 2020; Giorgio et al., 2020; London and Gallo, 2020) that could be repurposed for the treatment of musculoskeletal diseases/disorders or carcinomas. The drug-based therapeutics include kinase inhibitors, small molecules, monoclonal antibodies, antibody-drug conjugates, nanobodies and peptides that predominantly target either the kinase domain or the ligand binding domain of the receptor (Barquilla and Pasquale, 2015; Buckens et al., 2020). Depending on the target site, these approaches utilize either selective-agents, as demonstrated with the development of antibodies or less selective-agents such as kinase inhibitors (Giorgio et al., 2020). In the context of currently available drugs a number of pan-kinase inhibitor drug targets, Dasatinib, Sitravinib (MGCD516), JI-101, and XL647, and one selective drug target, an antibody targeting EphA3, Ifabotuzumab (KB004), are currently in clinical trials, predominantly Phase I trials (Buckens et al., 2020). More specific to the musculoskeletal field, a preclinical study treating osteosarcoma utilized drugs that inhibit receptor tyrosine kinase signaling, Pazopanib and Trametinib. The authors identified that this treatment down-regulated EphA2 and IL-7R, and silencing EphA2 resulted in significant reduction of cell proliferation and migration (Chiabotto et al., 2020).

Researchers are also developing novel strategies to identify and generate therapeutic targets with increasing specificity and efficiency. One such approach is the selection of Phage-displayed accessible recombinant targeted antibodies (SPARTA). This process utilizes *in vitro* phage-display screening followed by multiple rounds of sorting with yeast-display screening and the intravenous injection of the selected phage particles into tumor-bearing mice, where they undergo further selection, recovery and amplification (D'Angelo et al., 2018; Tang et al., 2020). This technique was used to generate anti-EphA5 antibodies that have shown specific targeting of EphA5 expressing lung cancer cells (D'Angelo et al., 2018; Tang et al., 2020). Another approach is the generation of peptide antagonists. The Eph-ephrin specific blocking peptides predominantly target the Eph receptor (Koolpe et al., 2002, 2005; Murai et al., 2003), with limited peptides targeting the ephrin ligands (Tanaka et al., 2010). The majority of these peptides bind to the ligand-binding domain of the Eph receptor limiting ephrin ligand binding and thus inhibiting Eph activation. Researchers have subsequently extended the half-life of existing peptides through the addition of polyethylene glycol polymer. One such example is TNYL-RAW, which blocks the binding of the ephrin ligand to the ligand binding domain of EphB4 (Noberini et al., 2011). Recently a peptide with dual function, specifically targeting EphB4-ephrin-B2 interactions was developed. This molecule, termed bi-directional ephrin agonist peptide (BIDEN-AP), can inhibit ephrin-B2 endothelial cell angiogenic signaling, while also activating EphB4 dependent tumor-suppressive signaling in tumor cells (Xiong et al., 2020). *In vivo* mouse studies confirm a significant reduction in ovarian tumor growth following the administration of BIDEN-AP (Xiong et al., 2020). This approach could be beneficial when targeting a known receptor-ligand pairing responsible for a specific biological function. Based on the current set of tools available to manipulate Eph-ephrin interactions, there is scope and potential for these therapeutic targets to be exploited and repurposed for the treatment of other diseases and disorders including those related to musculoskeletal pathophysiology.

## Concluding Remarks

There is considerable complexity in the intercellular interactions between numerous cell types within the bone microenvironment. Throughout this review we have highlighted the multifaceted Eph-ephrin interactions within and between stromal, hematopoietic and endothelial cell types and with the surrounding extracellular matrix. With the development of appropriate research tools including conditional knockout and transgenic mice, specialized *in vitro* culture systems, unique engineered substrates, soluble Eph and ephrin-Fc fusion proteins, Eph-ephrin inhibitory peptides and functional blocking antibodies, we have a greater understanding of how these cells interact within the bone through Eph-ephrin communication to maintain skeletal integrity. It is clear that the Eph-ephrin family members play a role in many vital biological processes during skeletal development and in maintaining skeletal physiology, where dysregulation can lead to a number of pathophysiological conditions within the musculoskeletal system. Eph-ephrin signaling research has identified potential new drug targets that could be exploited for the treatment of musculoskeletal conditions such as fracture repair, periprosthetic osteolysis or disease states such as osteoarthritis or osteoporosis.

Despite our extensive knowledge in this field, there is still a considerable amount of research required to fully understand the role of Eph-ephrin communication within the bone microenvironment. For example it is clear that the MSC population is highly heterogeneous. Could Ephs and ephrins be used as markers to identify MSC subsets? Certainly, this is already under investigation with the identification of EphA2 within different human MSC populations, and the proposed contribution of EphA5 regulating MSC growth, while numerous B-subclass members have been implicated in MSC niche maintenance. There is limited knowledge on the function of Eph-ephrin signaling within the marrow adipose tissue.

The involvement of numerous Eph-ephrin signaling partners contributing to a particular cellular process in a spatial and temporal manner is a reoccurring theme evident throughout this review. It is yet to be determined how multiple Eph receptors or ephrin ligands are expressed simultaneously over a range of developmental stages to differentially influence biological processes. Overall, Eph-ephrin interactions appear to be required as a mechanism to “fine tune” a myriad of processes required for skeletal development, maintenance and repair.

We are also just starting to appreciate the interaction of Eph-ephrin molecules with up-stream and down-stream signaling targets within resident cells of the bone microenvironment. Currently known targets include IGF-1 and PTH, which interact with Eph-ephrin signaling during osteoblast and osteoclast formation and function; the communication with CXCL12 signaling during hematopoietic niche maintenance; or the interaction with integrin molecules during cell adhesion of endothelial cells or osteoclasts. Knowing these molecular interactions and the associated up- or down-stream signaling pathways provides us with a better understanding not only of the biology but also the dynamics and fluidity that is required to develop potential therapeutic targets to treat musculoskeletal diseases and disorders. This is already evident with current therapeutic approaches targeting different domains of the Eph receptors and ephrin ligands to treat a range of cancers. Based on current knowledge there is an opportunity to combine and utilize multidisciplinary approaches to repurpose tools and drug targets to influence Eph-ephrin communication as a therapeutic strategy to treat diseases and disorders relating to musculoskeletal tissue.

## Author Contributions

AA and SG contributed to the writing and editing of the manuscript. All authors contributed to the article and approved the submitted version.

## Conflict of Interest

The authors declare that the research was conducted in the absence of any commercial or financial relationships that could be construed as a potential conflict of interest.
